# Comparing laser speckle contrast imaging and indocyanine green angiography for assessment of parathyroid perfusion

**DOI:** 10.1038/s41598-023-42649-2

**Published:** 2023-10-12

**Authors:** Emmanuel A. Mannoh, Naira Baregamian, Giju Thomas, Carmen C. Solόrzano, Anita Mahadevan-Jansen

**Affiliations:** 1 Vanderbilt Biophotonics Center, Vanderbilt University, PMB 351631, Nashville, TN 37235 USA; 2 Department of Biomedical Engineering, Vanderbilt University, Nashville, TN 37235 USA; 3 Division of Surgical Oncology and Endocrine Surgery, Vanderbilt University Medical Center, Nashville, TN 37232 USA; 4 Department of Surgery, Vanderbilt University Medical Center, Nashville, TN 37232 USA; 5 Department of Neurological Surgery, Vanderbilt University Medical Center, Nashville, TN 37232 USA; 6 Department of Otolaryngology, Vanderbilt University Medical Center, Nashville, TN 37232 USA

**Keywords:** Biomedical engineering, Translational research

## Abstract

Accurate intraoperative assessment of parathyroid blood flow is crucial to preserve function postoperatively. Indocyanine green (ICG) angiography has been successfully employed, however its conventional application has limitations. A label-free method overcomes these limitations, and laser speckle contrast imaging (LSCI) is one such method that can accurately detect and quantify differences in parathyroid perfusion. In this study, twenty-one patients undergoing thyroidectomy or parathyroidectomy were recruited to compare LSCI and ICG fluorescence intraoperatively. An experimental imaging device was used to image a total of 37 parathyroid glands. Scores of 0, 1 or 2 were assigned for ICG fluorescence by three observers based on perceived intensity: 0 for little to no fluorescence, 1 for moderate or patchy fluorescence, and 2 for strong fluorescence. Speckle contrast values were grouped according to these scores. Analyses of variance were performed to detect significant differences between groups. Lastly, ICG fluorescence intensity was calculated for each parathyroid gland and compared with speckle contrast in a linear regression. Results showed significant differences in speckle contrast between groups such that parathyroids with ICG score 0 had higher speckle contrast than those assigned ICG score 1, which in turn had higher speckle contrast than those assigned ICG score 2. This was further supported by a correlation coefficient of -0.81 between mean-normalized ICG fluorescence intensity and speckle contrast. This suggests that ICG angiography and LSCI detect similar differences in blood flow to parathyroid glands. Laser speckle contrast imaging shows promise as a label-free alternative that overcomes current limitations of ICG angiography for parathyroid assessment.

## Introduction

Preserving healthy parathyroid glands is of critical importance during endocrine neck surgeries. Failure to do so could result in an inability to produce sufficient levels of parathyroid hormone (PTH) for normal calcium regulation, termed hypoparathyroidism^1^. Consequently, the patient is likely to suffer from hypocalcemia – low levels of calcium circulating in the bloodstream. This causes increased neuromuscular irritability resulting in tingling, muscle cramps, cardiac arrhythmias and seizures^1,2^. Reports on the incidence of hypoparathyroidism after thyroidectomy vary based on surgeon experience and disease definition^3–5^. However, a review of 115 studies found the rates of temporary and permanent hypoparathyroidism to be between 19–38 and 0–3% respectively (interquartile ranges)^6^. Reducing these rates is important, as treatment of hypoparathyroidism is sometimes associated with negative side effects. Conventional treatment involves supplementation with calcium and activated vitamin D, which, if required long term, can lead to nephrocalcinosis, kidney stones, and brain calcifications^1,7^. Replacement therapy with PTH is an alternative, however further studies are required to determine if it reduces the risk of long-term complications^8^. There is a need, therefore, to develop tools that provide guidance to surgeons intraoperatively to help reduce the incidence of post-surgical hypoparathyroidism.

There are two main challenges surgeons face in the preservation of healthy parathyroid glands during surgery. The first involves identification of the glands. Due to their small size^9^, visual similarity to other tissues such as lymph nodes^10^, and variability in location^11,12^, intraoperative localization of parathyroid glands can be challenging. Near-infrared autofluorescence (NIRAF) detection has emerged as a reliable technique to localize or confirm identification of parathyroid glands intraoperatively^13–23^. There are currently two clinical devices with U.S. Food and Drug Administration clearance and Conformité Européenne marking for parathyroid identification using NIRAF detection^24^. However, identification alone is insufficient to improve rates of post-surgical hypoparathyroidism^25–28^.

The second challenge involves preserving the blood supply to the parathyroid glands and accurately assessing their perfusion status at the end of the surgery. Correctly assessed, the function of a devascularized parathyroid gland (i.e. a gland whose blood supply has been completely damaged) can be salvaged by autotransplantation^29^. Recently, indocyanine green (ICG) angiography has emerged as a promising technique to aid in preservation of the parathyroid blood supply and evaluating the parathyroids’ perfusion status^30–34^. It has been reported that a minimum of one parathyroid gland with strong ICG fluorescence is sufficient for normal postoperative parathyroid function^30^, though other studies report there is no benefit to the technique^35^. Part of the reason for this discrepancy is that the current method relies on qualitative scoring of ICG fluorescence intensity^30^. Furthermore, the timeline of imaging is extremely important as variation in timing between ICG injection and commencement of imaging could result in inaccurate scores. Other limitations to the technique include the fact that it cannot be simultaneously combined with NIRAF detection for parathyroid identification, and the possibility for severe allergic reactions to the dye^36^. These limitations would be overcome by a label-free method to assess parathyroid perfusion.

Laser speckle contrast imaging (LSCI) is a label-free approach that has shown success in evaluating blood flow to parathyroid glands^37^. Intraoperative measurements using this technique have been shown to be strongly related to postoperative outcomes of total thyroidectomy patients^38^. The technique analyzes blurring of the speckle pattern produced when coherent laser light illuminates a tissue surface, providing contrast between regions of varying blood flow. Speckle contrast is calculated as the standard deviation of pixel intensities within local regions of the speckle pattern, divided by their corresponding mean intensities, with smaller values indicating greater blood flow^39^.

The purpose of the proceeding study is to examine the relationship between intraoperative LSCI and ICG angiography of the same parathyroid glands. Speckle contrast measurements were compared against ICG scores and fluorescence counts, to determine whether the methods make similar assessments of parathyroid perfusion.

## Results

**Figure 1:**
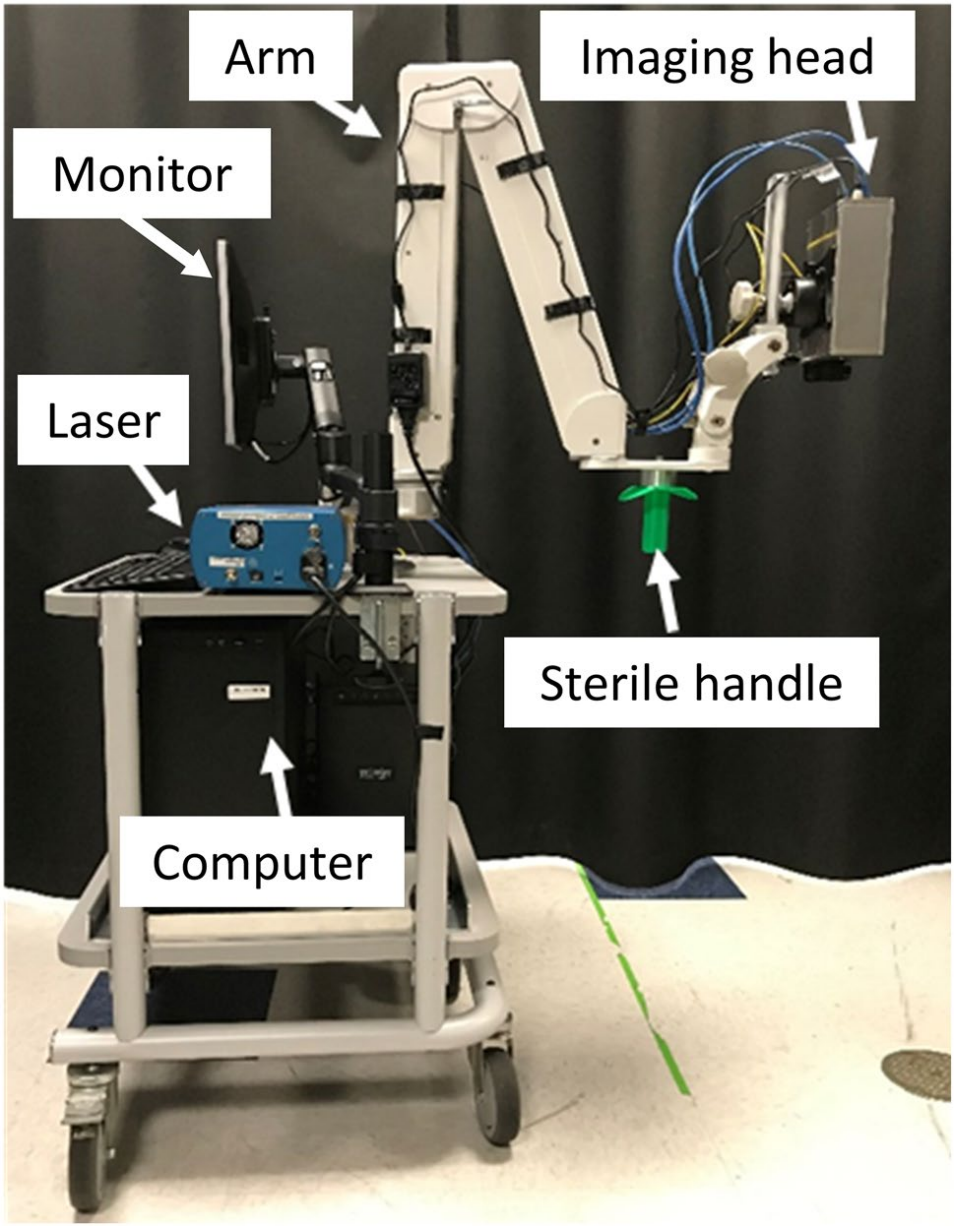
Image of the parathyroid speckle and autofluorescence imager (ParaSPAI).

**Figure 2:**
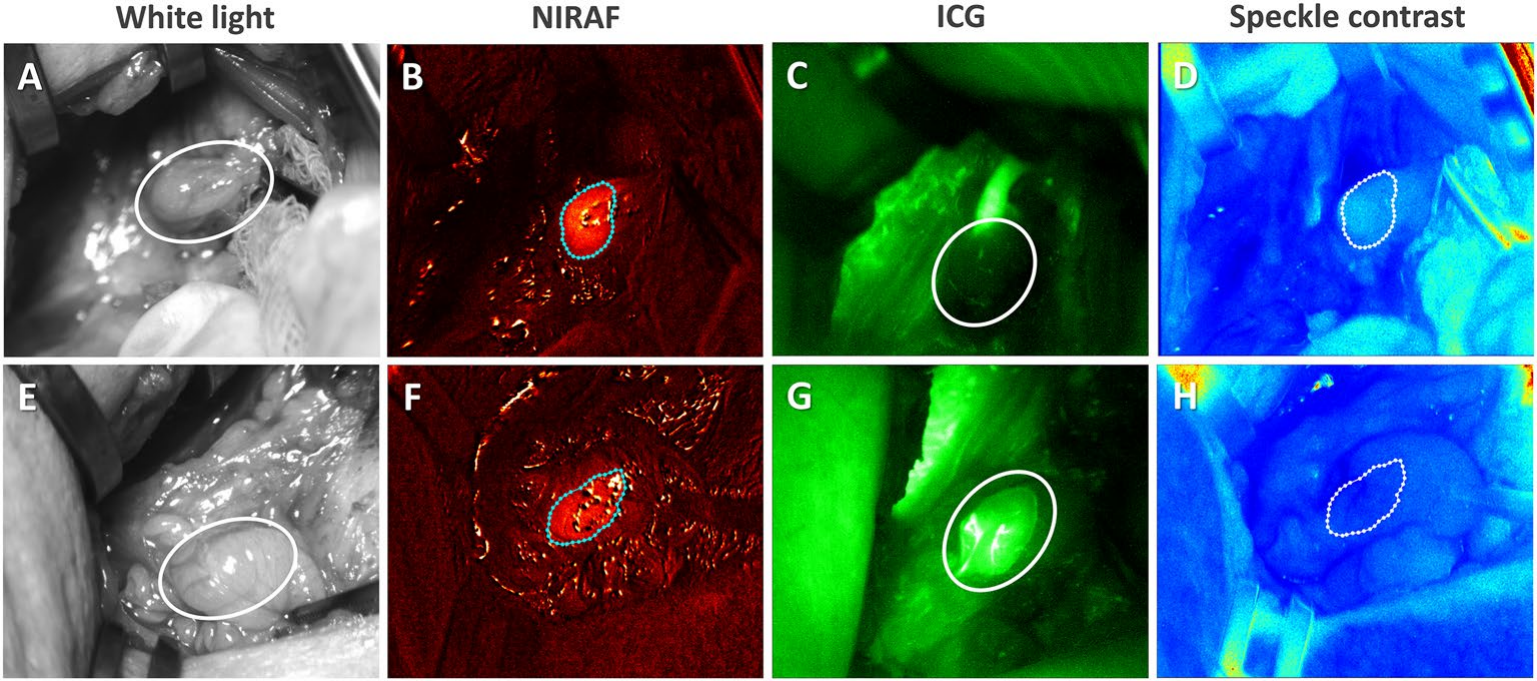
Representative white light, near-infrared autofluorescence (NIRAF), indocyanine green (ICG) fluorescence, and speckle contrast images of a devascularized (**A**–**D**) and a well-perfused (**E–H**) parathyroid gland, acquired with the imaging device. The parathyroid glands are indicated with white ellipses in white light and ICG fluorescence images, and with dotted contours generated by automated segmentation in NIRAF and speckle contrast images.

Thirty-seven parathyroid glands from 21 different patients were included in this study. Label-free imaging (LSCI & NIRAF) and ICG imaging were performed using a previously developed parathyroid speckle and autofluorescence imager (ParaSPAI)^40^, shown in Fig. 1. Representative images of a well-perfused and a devascularized parathyroid gland are displayed in Fig. 2. Both sets of images were acquired in a thyroid lobectomy case.

### Comparing ICG scores and speckle contrast

**Figure 3:**
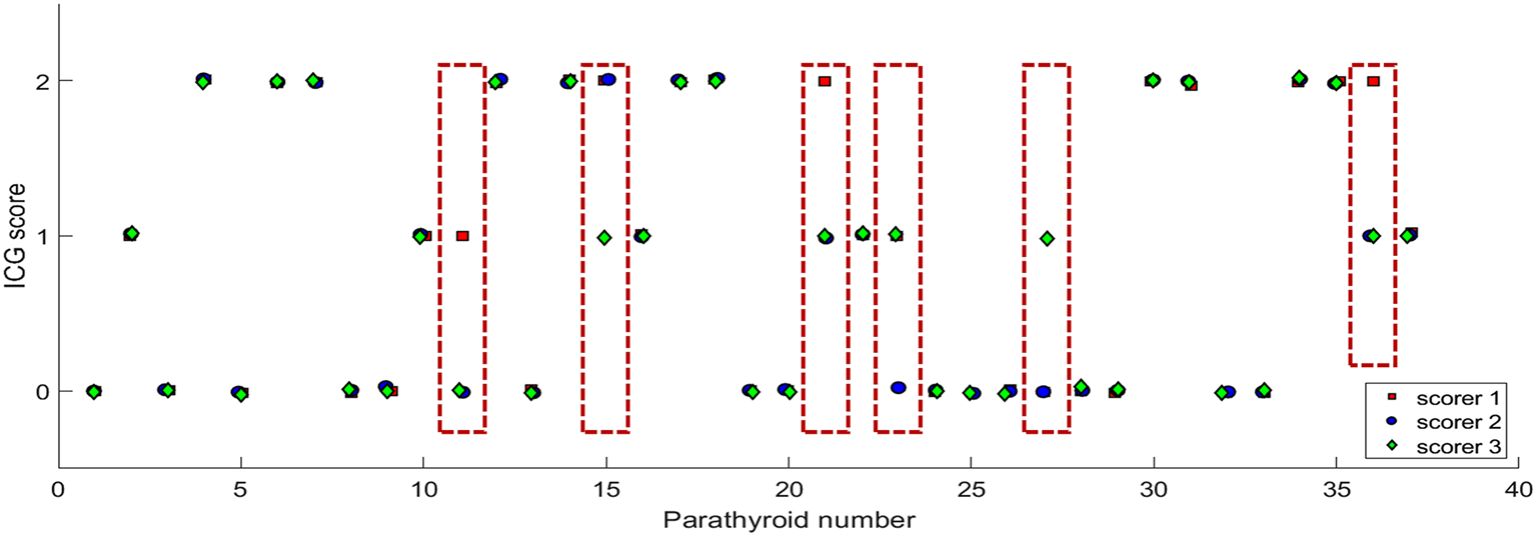
Indocyanine green (ICG) scores assigned to 37 parathyroid glands imaged intraoperatively, assigned by 3 different scorers. Red boxes indicate instances of disagreement between the scorers. These all involve discrepancies in assigning ICG score 1.

**Figure 4:**
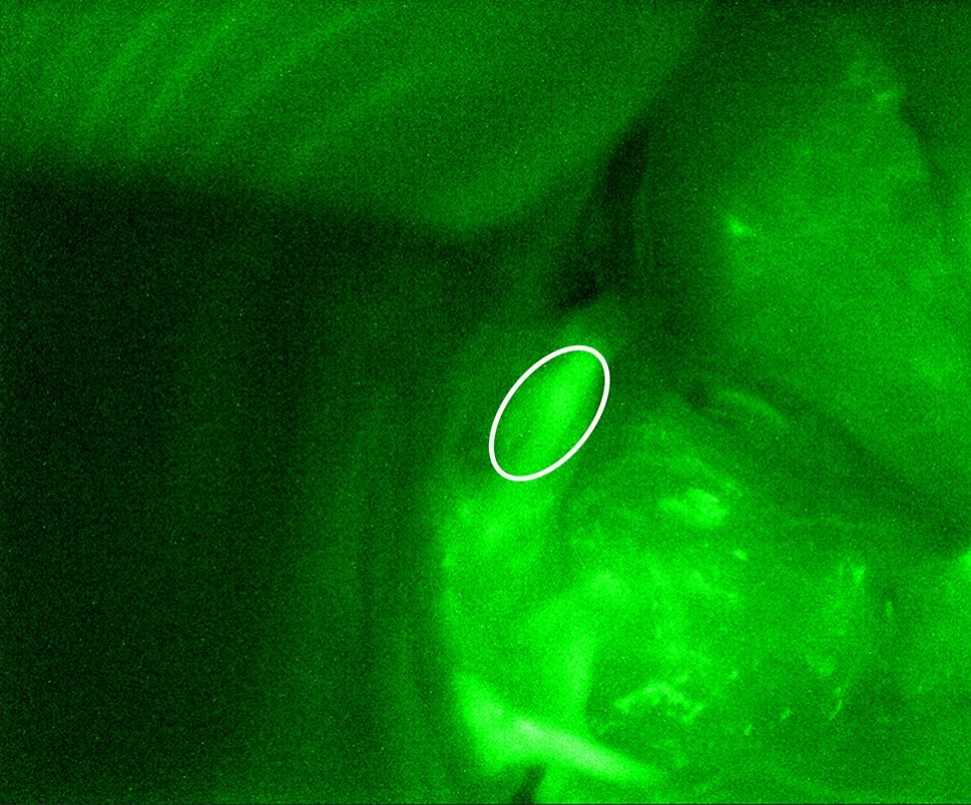
An example indocyanine green (ICG) fluorescence image showing a parathyroid gland that received differing ICG scores. One scorer assigned a score of 2, and the other two assigned a score of 1.

Three scorers (two research personnel and one surgeon) independently assigned scores of 0, 1, or 2 to each parathyroid gland based on their ICG fluorescence. The distribution of ICG scores assigned to each gland by the three scorers is shown in Fig. 3. All scorers were in agreement for 31 (83.8%) of the glands. The exceptions are indicated within dashed red boxes in Fig. 3 and always involve a discrepancy with assigning an ICG score of 1 (which denotes moderate or patchy ICG fluorescence). In fact, of the 11 parathyroid glands that received an ICG score of 1 from any scorer, only 5 had all scorers in agreement. An example of a parathyroid gland that received disparate scores is shown in Fig. 4, indicated by a white ellipse. One scorer assigned a value of 2, another scored it as 1, and the third scored it as being between 1 and 2 (1 was chosen to fit with the 3-level scoring system). There were no instances where one scorer assigned an ICG score of 0 and another, an ICG score of 2 for the same gland.

**Figure 5:**
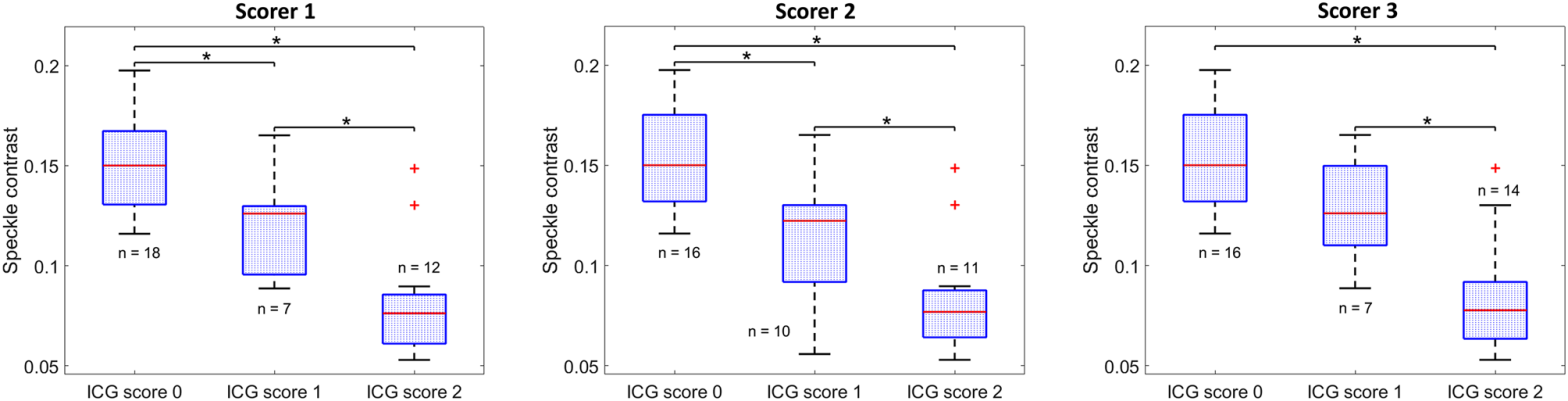
Boxplots showing distribution of speckle contrast for parathyroid glands assigned indocyanine green (ICG) scores 0, 1 and 2 by three different scorers. Each box represents the interquartile range for data in that group, the red horizontal line indicates the median for the group, whiskers extend to the most extreme data point not considered an outlier, and outliers are represented by red crosses. Significant differences (p < 0.05) between groups are indicated with black asterisks (*).

Following ICG scoring, average speckle contrast for each of the 37 parathyroid glands was grouped according to the ICG scores provided by each scorer. A two-way ANOVA on this grouped speckle contrast data revealed that, regardless of the differences observable in Fig. 3, overall there was no significant influence of the scorer on the model (p = 0.77). Additionally, the interaction term assessing interaction between ICG score and scorer was not statistically significant (p = 0.98). The influence of ICG score was however significant (p < 10^–4^). This meant that the ICG score of a parathyroid gland was related to its speckle contrast in a statistically significant manner, and that this relationship was not influenced by the choice of scorer. Consequently, the data was reanalyzed disregarding the influence of the scorer and interaction terms. Three separate one-way ANOVAs were performed for each scorer, followed by multiple comparisons between ICG scores. The results are shown as the boxplots in Fig. 5.

For scorers 1 and 2, all groups had significantly different speckle contrast from one another. For scorer 3, the speckle contrast of parathyroid glands assigned ICG score 0 were significantly different (p < 0.05) from the speckle contrast of parathyroid glands assigned ICG score 2. Similarly, the speckle contrast of parathyroid glands assigned ICG score 1 were significantly different from those assigned ICG score 2. However, there was no significant difference in speckle contrast between ICG score 0 and ICG score 1. For all scorers, there is a notable trend where speckle contrast is lower for higher ICG scores. This makes sense since lower speckle contrast and higher ICG score both indicate greater perfusion.

### Comparing ICG fluorescence intensity and speckle contrast

**Figure 6:**
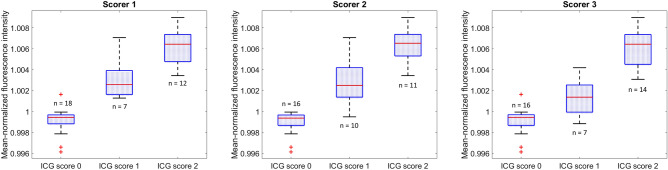
Boxplots showing distribution of mean-normalized fluorescence intensity for parathyroid glands assigned indocyanine green (ICG) scores 0, 1 and 2 by three different scorers. Each box represents the interquartile range for data in that group, the red horizontal line indicates the median for the group, whiskers extend to the most extreme data point not considered an outlier, and outliers are represented by red crosses.

**Figure 7:**
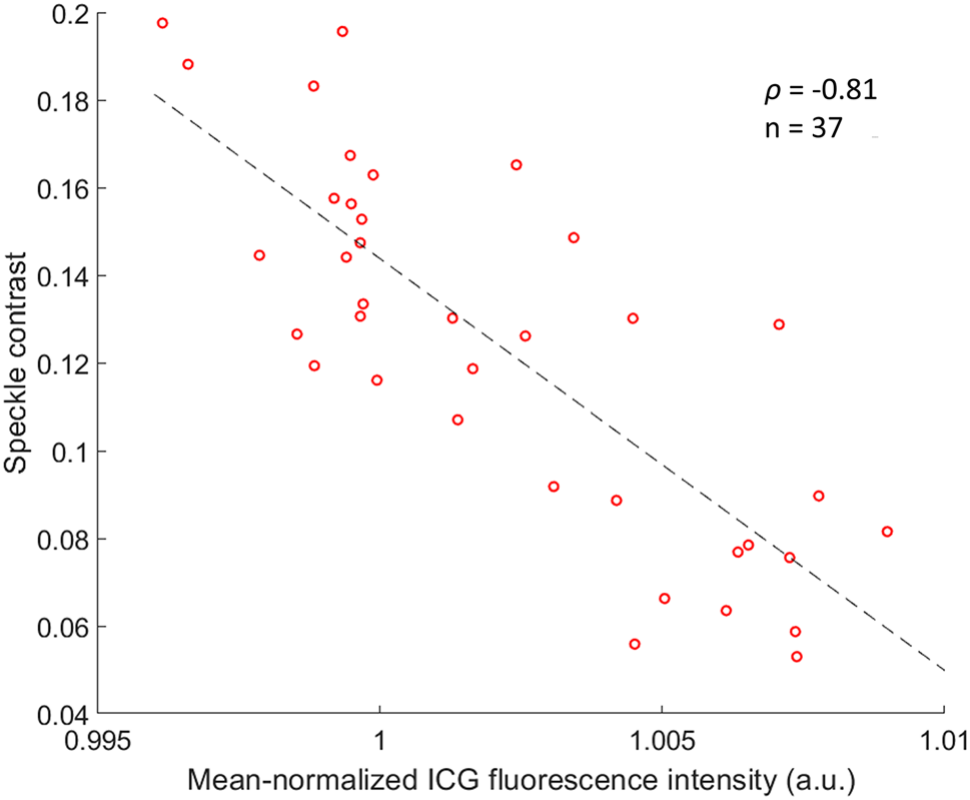
Scatterplot of speckle contrast of 37 parathyroid glands against their respective mean-normalized indocyanine green (ICG) fluorescence intensity. The line of best fit relating the two quantities is indicated by the dashed line. A linear correlation coefficient of − 0.81 was obtained, suggesting strong similarity between the two techniques.

To compare LSCI and ICG angiography more quantitatively, the ICG fluorescence intensity was calculated for each parathyroid gland. This was possible due to the fixed imaging distance for all data acquisition. To account for variability in patient anatomy influencing the amount of ICG reaching the surgical field, and variability in the timing of imaging, each fluorescence intensity value was normalized to the mean fluorescence intensity across the entire image it was extracted from. The distribution of mean-normalized fluorescence intensity according to ICG scores is shown in Fig. 6. As would be expected, fluorescence intensity is higher for larger ICG scores. Figure 7 shows the speckle contrast of all 37 parathyroid glands, plotted against their mean-normalized ICG fluorescence intensity. A linear correlation coefficient of -0.81 relating the two measures was obtained from analysis. This implies a close-to-linear relationship in which speckle contrast decreases as ICG fluorescence intensity increases.

## Discussion

Accurate assessment of blood flow to the parathyroid glands is important for ensuring postoperative parathyroid functional preservation and normocalcemia in patients after endocrine neck surgeries. Currently, the assessment is primarily made by visual inspection of the glands and confirmation of bright red bleeding after needle prick or small cut of the parathyroid^41^. Recently, ICG angiography has emerged as a promising technique to help improve the accuracy of surgeons’ assessments of parathyroid perfusion. However, the technique has some limitations, such as the inability to be used simultaneously with NIRAF detection. The fluorescence spectrum of ICG overlaps with that of the parathyroid autofluorescence signal^42^, and is much stronger in intensity. Therefore, once ICG has been administered in a case, NIRAF can no longer be used to identify parathyroid glands. This limitation, along with the possibility for allergic reactions and subjectivity in scoring could be avoided with the use of a label-free quantitative method such as LSCI.

In this study, we compared intraoperative speckle contrast with ICG scores and ICG fluorescence intensity for 37 parathyroid glands in 21 thyroidectomy and parathyroidectomy patients. Using a previously-developed combined fluorescence and speckle imaging device^40^, both sets of data could be acquired for the same parathyroid glands using the same instrument. While a parathyroid adenoma is clinically very different from a healthy parathyroid gland, data from the two types of glands could be combined in this study because the goal was to compare perfusion assessments made by one method with another, with no influence on or assessment of patient outcome.

Three observers scored the ICG images while blinded from each other’s scores and speckle contrast data. The results highlight the ambiguity in assigning an ICG score of 1 in a qualitative 3-level system. For instance, the parathyroid gland in Fig. 4 received a score of 2 from one scorer, and a score of 1 from the other two. Imagining a scenario where this parathyroid gland was the best perfused or only gland identified after a total thyroidectomy, its ICG score could influence decisions made by the surgeon. In a study involving 36 thyroidectomy patients, Fortuny et al.^30^ reported normal postoperative PTH levels for all patients in whom at least one parathyroid gland with an ICG score of 2 was identified. Depending on the scorer, the patient in the above hypothetical scenario could either be expected to have normal or low postoperative PTH. It is possible that similar ambiguities in scoring contributed to the findings by Razavi et al*.*^35^ who report that parathyroid ICG scores are not associated with post-thyroidectomy patient outcomes.

For all scorers, the median speckle contrast of parathyroid glands assigned ICG score 0 was higher than the median of parathyroids assigned ICG score 1, which in turn was higher than the median of parathyroids assigned ICG score 2. Smaller speckle contrast values indicate greater blood flow, therefore this trend is expected. In all but one instance, these differences in speckle contrast across the ICG score groups were statistically significant, suggesting that LSCI and ICG angiography detect similar differences in blood flow to parathyroid glands. Outliers in Fig. 5 represent parathyroid glands which were deemed to have strong ICG fluorescence but had much higher speckle contrast than other glands placed in this group. A possible explanation could be a leak (bleeding) that contaminated these glands with ICG, making them strongly fluoresce even though they may not have been well-perfused. A larger study that also examines postoperative outcomes is needed to help better understand scenarios like these.

To more quantitatively evaluate the similarity between the two techniques, the average fluorescence intensity of each parathyroid gland was calculated from ICG images. Quantification of ICG fluorescence with handheld cameras currently on the market is challenging as variability in distance to the target and angle of imaging result in variability of fluorescence intensity^43^. With the imaging system used in this study, this problem was minimized. The results revealed a strong negative correlation between speckle contrast and mean-normalized ICG fluorescence intensity, thus corroborating our findings that the two techniques provide similar information. Further supporting the similarity of the two techniques is the fact that two previous studies, one using ICG angiography^30^, and the other using LSCI^38^, both concluded that a minimum of one well-perfused parathyroid gland is needed for normal parathyroid function after total thyroidectomy.

While there are similarities in the results of parathyroid assessment using the two techniques, it is important to note relevant differences. ICG angiography detects the presence of blood containing the dye, while LSCI simply detects the flow of blood. For this reason, if imaging is timed appropriately, the arterial supply to a parathyroid gland can be unequivocally highlighted after an injection of ICG – the fluorescence of the dye can be tracked as it enters the parathyroid gland. This can be extremely useful early on in the operation to alert the surgeon to the location of the blood vessel and help them avoid damage to it. Since LSCI detects the flow of all blood, while it might highlight blood vessels, it will not provide an indication as to which blood vessel is directly supplying the parathyroid. Additionally, while fluorescence may be visible underneath a few millimeters of tissue, LSCI is very superficial (this does not prevent being able to evaluate the surface perfusion of parathyroid glands). Work is ongoing to further develop the technique so as to be able to image deeper blood vessels^44^. Since LSCI analyzes light scattering due to motion, it is also more susceptible to bulk motion artifacts which might alter readings whereas a fluorescence image might just get blurry. Efforts have been made to overcome this problem with LSCI such as by monitoring fiducials with high speckle contrast^45,46^, and using image registration^47,48^.

Another result of the difference between the two methods is that ICG angiography is susceptible to false positives if a blood vessel is damaged after injection of the dye, while LSCI is not. If a blood vessel is cut and the dye leaks out, it could result in the entire surgical field fluorescing, giving the impression of adequate perfusion. Furthermore, since the dye persists for several minutes^30^, damage to a previously well-perfused gland will not be detectable until the fluorescence levels have dropped low enough for a subsequent ICG injection. Due to its label-free nature, LSCI also has an advantage over ICG angiography in that it can be performed multiple times throughout surgery without the risk of increased toxicity. The intensity and wavelength of light used in this study pose no harm to the patient. Finally, LSCI can be seamlessly integrated with NIRAF detection for parathyroid localization (as was demonstrated in the development of the ParaSPAI^40^), whereas NIRAF detection is no longer possible after ICG angiography. While LSCI shows promise, further investigation is needed to understand the implications of intraoperative decision-making based on the technique. Work on this goal has already begun with a study showed that total thyroidectomy patient outcomes are strongly related to intraoperative LSCI measurements^38^.

In this study, we demonstrated that speckle contrast correlates strongly with ICG fluorescence in the same parathyroid glands intraoperatively. While ICG scoring can be ambiguous, LSCI provides objective quantitative values on blood flow to a parathyroid gland. It also allows seamless integration with NIRAF detection to provide complete assessment of parathyroid glands in endocrine neck surgeries by confirming the presence of parathyroid tissue and its perfusion. Laser speckle contrast imaging is a promising label-free alternative to ICG angiography for intraoperative parathyroid perfusion assessment.

## Methods

### Patient recruitment and imaging protocol

This study was approved by the Vanderbilt University Medical Center Institutional Review Board. All methods were performed in accordance with relevant guidelines and regulations. Twenty-one patients (17 female, 4 male) undergoing partialthyroidectomy (7), total thyroidectomy (6), or parathyroidectomy (8) were recruited and written informed consent was obtained from each patient prior to participation. Label-free imaging (LSCI & NIRAF) and ICG imaging were performed using a previously developed parathyroid speckle and autofluorescence imager (ParaSPAI)^40^, shown in Fig. 1. The ParaSPAI is an investigational device developed for research purposes. While imaging was performed with the room lights on, the overhead OR lamps and the surgeon’s headlamp had to be pointed away from the surgical field. The imaging procedure depended on the type of surgery being performed. In thyroidectomy cases, imaging began after the thyroid lobe or total thyroid had been excised. The attending surgeon positioned the device above each parathyroid gland they identified during the operation and label-free images were acquired. Camera exposure times for LSCI and NIRAF were 5 ms and 100–300 ms, respectively. Following this, 1 mL of a 2.5 mg/mL ICG solution was administered intravenously, followed by a saline flush. With the same device still positioned above a parathyroid gland, ICG fluorescence images were recorded after the saline flush. Images were obtained until the ICG fluorescence intensity peaked and plateaued or began to gradually decrease (20–30 s after saline flush). At this point, the device was then quickly positioned above any other parathyroid glands to record at least 5 s of data for each.

In parathyroidectomy cases, imaging began after the diseased parathyroid gland had been localized by the attending surgeon. Before ligating the parathyroid blood supply in preparation for excision, label-free imaging of the diseased gland was performed. Next, the parathyroid blood supply was ligated and label-free images of the diseased gland were again acquired. Additionally, any other unligated parathyroid glands identified by the surgeon were then imaged. After label-free imaging, 1 mL of a 2.5 mg/mL ICG solution was administered intravenously, followed by a saline flush. ICG fluorescence images were recorded as described for thyroidectomy procedures. The imaging (label-free plus ICG) added no more than 10 min to the surgery duration for each patient and no repeat ICG injections were performed. In order to avoid influencing patient care, the attending surgeon was blinded from images acquired by the device intraoperatively. Qualitative scoring of ICG fluorescence intensity was performed postoperatively and is discussed in detail below.

### Data analysis

A total of 37 parathyroid glands were imaged in this study. Of these, 9 were adenomas while the remaining 28 were normal. For each parathyroid gland, an image was selected from the ICG recording (after peaking of the fluorescence intensity) and a qualitative score of fluorescence intensity was independently assigned by three different scorers. The scorers were two research personnel and one of the surgeons that participated in the study, all of whom had experience with NIRAF, LSCI and ICG, and were blinded from each other’s scores. The scoring system used was similar to that reported by Fortuny et al*.*^30^ in their demonstration of ICG angiography for assessment of parathyroid viability. A parathyroid gland with little to no ICG fluorescence received a score of 0, a parathyroid gland with moderate or patchy ICG fluorescence received a score of 1, and a parathyroid gland with strong ICG fluorescence received a score of 2. The speckle contrast data was grouped according to these ICG scores for each observer. A two-way analysis of variance (ANOVA), followed by multiple comparisons testing was performed on the three groupings to identify any significant differences in speckle contrast between the groups as identified by ICG scores and scorers. The tests were performed using MATLAB software (The MathWorks Inc., Natick, MA).

Given that the imaging system used in this study acquires images with a fixed distance between camera and subject, fluorescence intensity can be quantified instead of simply assigning qualitative scores. This quantification was performed by manually segmenting the ICG fluorescence images and calculating the average intensity for each parathyroid gland. In order to account for patient-to-patient variability in the amount of ICG entering the surgical field, as well as variability due to ICG draining from the surgical field, the average intensity of each parathyroid gland was normalized to the average intensity for that entire image. This mean-normalized fluorescence intensity was then compared to the parathyroid speckle contrast in a regression, and a linear correlation coefficient was calculated.

## Data Availability

The datasets generated and analysed during the current study are available from the corresponding author on reasonable request.

## Abbreviations

ANOVA: Analysis of variance
ICG: Indocyanine green
LSCI: Laser speckle contrast imaging
NIRAF: Near-infrared autofluorescence
ParaSPAI: Parathyroid speckle and autofluorescence imager
PTH: Parathyroid hormone
